# “You get stuck in it”: Young people's accounts of attempting to quit non-medical tramadol use

**DOI:** 10.1177/14550725231160330

**Published:** 2023-04-02

**Authors:** Kristin Arve

**Affiliations:** 341565 Lund University School of Social Work, Lund, Sweden

**Keywords:** non-medical prescription opioid use‌, tramadol, young people‌, recovery, drug treatment‌

## Abstract

**Aim:** Non-medical use of tramadol and other prescription opioid use has become a great concern in many countries, including Sweden. This study examines key components in young people's accounts of attempting to quit drugs, focusing on non-medical use of tramadol. **Methods:** Repeated qualitative interviews were conducted with 12 individuals aged 19–24 years with experiences of problems related to non-medical tramadol use. The analysis used the concepts of autonomy, competence, and relatedness from self-determination theory. **Results:** Three themes emerged from the young people's accounts: (1) quitting initiated by parents and professionals; (2) being willing, but unable; and (3) between ambivalence and determination. These themes demonstrate conflicting emotions towards drug use along with a significant external pressure to quit, but also difficulties in quitting due to experiences of dependence, withdrawal symptoms, and mental health issues. For most participants, however, an increasing autonomous will and ability to abstain from drugs gradually developed, with the support from trusted relationships with professionals, family, and friends playing a crucial role. **Conclusion:** The process of trying to quit non-medical tramadol use can be challenging and involve a complex interaction between willingness and capability, where external influence can be either facilitating or hindering. This study highlights the importance of taking into account young people's own perspectives in treatment efforts, where trust is a key component.

## Introduction

Non-medical use of prescription opioids has become a growing concern globally. In Sweden, tramadol is the opioid most commonly used and it represents a novel pattern of substance use among young people (Holmstedt et al., 2020; Olsson et al., 2017; Richert & Johnson, 2013). Studies also suggest that non-medical use of tramadol is a recognised concern in other Nordic countries, such as Finland, as well as in regions such as West Africa and the Middle East (Bashirian et al., 2014; Bassiony et al., 2015; Fuseini et al., 2019; Häkkinen et al., 2014; Ibrahim et al., 2017).

Tramadol is a synthetic opioid used for pain relief that, unlike other opioids, also has both activating and antidepressant effects (Holmstedt et al., 2020; Svensson & Karlsson, 2018). Previous studies show that people use tramadol to feel euphoric, relaxed, and to relieve emotional distress, but also to stay alert or enhance sexual performance (Bassiony et al., 2015; Finsam, 2019; Fuseini et al., 2019; Ibrahim et al., 2017). In addition to the desirable effects of the drug, tramadol is perceived as relatively easily accessible and “safer” compared to other opioids (Richert & Johnson, 2013; Svensson & Karlsson, 2018). Historically, tramadol was considered to have fewer adverse effects than other opioids, but since the early 2000s, the risks of dependence, seizures, and death have received more attention (Boostani & Derekhsan, 2012; Shadnia et al., 2008; Tjäderborn et al., 2007). Non-medical use of tramadol also often involves significantly higher doses than medical use, which increases the risks of side effects (Svensson & Karlsson, 2018).

Previous research on non-medical use of tramadol has primarily employed a quantitative approach and has concentrated on the prevalence of use, the characteristics of users, and the effects of the drug. However, there is limited knowledge about young people's own perspectives on their tramadol use and the process of trying to quit the drug, which will be addressed in this article. The process of trying to quit using drugs involves complex interactions between multiple factors (Bacchus et al., 2000). Although some aspects are more generalised, there are also differences between subgroups of drug users. Tramadol differs greatly from cannabis, which is the dominant drug of choice among young people. Regular use of tramadol causes withdrawal symptoms similar to other opioids (Ibrahim et al., 2017). In comparison with other opioids, however, tramadol enters the drug career of many young users early on (Holmstedt et al., 2020; Richert & Johnson, 2013). A study carried out at an outpatient clinic in Sweden shows a median age of onset of tramadol use of 16 years (Holmstedt et al., 2020).

Furthermore, there are factors that differentiate young drug users from older drug users, including developmental aspects and shorter drug use history (Wilson & Saggers, 2013). It is known that young adults—those aged 18–25 years—use drugs to a greater extent than other age groups (Alley et al., 2014; Arnett, 2005; Rodriguez & Smith, 2014). This age is characterised by identity development and opportunities, but also by instability and a feeling of being “in between” adolescence and adulthood (Arnett, 2005). Several researchers claim that young people generally have less motivation to stop using drugs and undergo treatment, as they have not yet experienced as many negative consequences from their drug use (Battjes et al., 2003; Chan et al., 2004). They also often initiate treatment due to external pressure and demands from social services, parents, or the judiciary (Battjes et al., 2003; Hugo et al., 2021; Wilson & Saggers, 2013). Young people also have a higher rate of dropping out of treatment (Rodriguez & Smith, 2014). In addition, a study from an outpatient clinic in Sweden found that treatment dropout rates were significantly higher among tramadol users and individuals aged 18–25 years, compared to other young drug users (Herrnsdorf et al., 2022).

Drug problems often occur alongside various forms of mental health issues. Drug use can serve as self-medication for underlying mental disorders, but also affect the brain in a way that may increase psychiatric symptoms (Grubb, 2019; Richert et al., 2020). The relationship between mental illness and substance use can be described as dynamic, as they reinforce each other (Tengström & Gunnarsson, 2012). Withdrawal symptoms from tramadol and other opioids also include psychiatric symptoms, such as depression, which can persist for a long period of time (Johnson et al., 2017; Finsam, 2019).

Another central aspect in the process of trying to quit drugs are interpersonal relationships. Supportive relationships can be encouraging in the recovery process, while drug-using friends are considered a risk factor for relapse (Nash et al., 2015; Rodriguez & Smith, 2014). Young people often experience conflicting feelings between wanting to continue hanging out with friends and an awareness of the associated risks (Mason et al., 2008; Rodriguez & Smith, 2014). Foster and Spencer (2013) point out that drug use with friends is also intertwined with feelings of intimacy, belonging, and sharing something together, especially for marginalised groups who often lack other trusting relationships. Herold and Søgaard (2019) challenges the simplification of drug-using friends as simply “bad company” and highlights that they can sometimes also be integrated into the recovery process.

In summary, there are both drug-specific, individual, and contextual factors that interact in the process of trying to quit drugs. The aim of the present study was to examine key components in young people's accounts of trying to quit drugs, with a focus on non-medical use of tramadol. The article focuses on identifying internal and external circumstances that they mention as facilitating or hindering in their attempts to quit the drug, with special attention to experiences of treatment interventions.

## Theoretical approach

Motivation is a critical aspect in all change processes, including quitting drugs. In the analysis of the young people's accounts, concepts from self-determination theory are applied. This theory distinguishes between intrinsic/autonomous and external/controlled motivation (Deci & Ryan, 2008). Intrinsic motivation refers to acting on the basis of one's own autonomy and will, where positive emotions stem from the behaviour itself. External motivation, on the other hand, connotes acting based on forces outside oneself, such as to receive a reward or avoid a punishment. This can, in turn, undermine intrinsic motivation due to the experience of feeling controlled (Deci & Ryan, 2008).

Self-determination theory emphasises the importance of the sociocultural context for motivation. Behaviours that are initially externally motivated can also be internalised and integrated into the persons sense of self. However, this requires a social context where the basic psychological needs of autonomy, competence, and relatedness are met (Deci & Ryan, 2008). *Autonomy* can be described as the experience of acting on the basis of one's own will, in accordance with one's own genuine interests and values. *Competence* refers to the person's ability to act and make changes in important areas of life. *Relatedness* can be described as an experience of connection with other people, which includes both the experience of being cared for and the feeling of being important to others. Ryan and Deci (2017) state that the dynamics between these needs are fundamental in the internalisation process from external to internal motivation. An environment that hinders the satisfaction of these needs, however, can have a negative impact (Deci & Ryan, 2008).

Self-determination theory has been applied in several previous studies to examine the motivation to participate in treatment and quit drugs (Hugo et al., 2021; Ryan et al., 1995; Wild et al., 2006). While external motivation in general is associated with poorer outcomes, there are also situations where external influences can promote internal motivation. Emotionally supportive relationships can create space for critical reflection about drug use, which can be crucial in initiating a change process (Goodman et al., 2015). A study by Hugo et al. (2021) found that willingness to change was often developed in dialogue with the therapist. They describe this as an internalisation process where the therapist becomes a bridge-builder between external and internal motivation.

## Research methods

A qualitative study was carried out using semi-structured interviews with 12 individuals aged 19–24 years who had had experiences with non-medical use of tramadol. There are different definitions of the term “young people”; in this study a delimitation has been made to those aged 18–24 years. The inclusion criteria for the participants was that they had experienced problems related to tramadol use. The research project was approved by the Swedish Ethical Review Authority (Dnr 2020-04519). The majority of participants were recruited through staff at treatment institutions and outpatient clinics in southern Sweden. In addition, two participants without treatment experience were recruited through other contacts, one through another participant and one through a colleague of the researcher.

The interviews were conducted during 2021. All but three of the participants were interviewed two, or in some cases, three times each, with a few months in between. The reason for this was the possibility to obtain richer data, as noted by Hydén (2000). In total, 23 interviews were conducted: 13 interviews were conducted face-to-face; seven were online and video-based; and three were conducted over the phone. The mode of interviewing was based on practical considerations and the preferences of the participants. Both video-based interviews and telephone interviews have been found to be useful in qualitative research and are considered to be relatively equivalent to face-to-face interviews (Archibald et al., 2019; Trier-Bieniek, 2012). The interviews lasted 28–64 min. The participants were asked about their history of drug use, with special attention to their use of tramadol, as well as their experiences of treatment. The interviews were recorded and transcribed verbatim. To protect the anonymity of the participants, each of them were given a pseudonym, and names and places that could identify them have been changed.

The data was analysed using thematic analysis. The interview transcripts were read multiple times in order for the researcher to become familiar with the data. After that, the material was coded in order to identify patterns and meaning, create structure, and reduce the amount of data (Rennstam & Wästerfors, 2011; Terry et al., 2017). Interviews from each participant were also studied individually to gain an overall understanding of their accounts, with a focus on key events. The concept of “accounts” is used to describe the young people's experiences, including both more descriptive and interpretive elements. The analysis was iterative, with themes gradually becoming more clearly defined (Terry et al., 2017). In this report, some small adjustments have been made to the selected quotes to make the text more readable.

## Participant characteristics

The participants consisted of 12 individuals (10 men, 2 women; age range = 19–24 years). Difficulty was encountered in recruiting female participants, which may be related to the general predominance of male drug users (EMCDDA, 2021). The participants’ use of tramadol was in the range of 1.5–5 years, and most of them had used the drug more or less on a daily basis. All but one participant stated that they had experienced a dependence on tramadol. All participants had a history of polydrug use, and tramadol had been one of their primary drugs. Half of the participants eventually changed their main drug, four to stronger opioids such as heroin. However, all participants had experiences of trying to quit tramadol, even if they later mainly used other drugs. Three of the young people had been drug-free for 1–2 years at the time of the interviews, while the others had more recently quit drugs or had had relapses. Only one participant was still using tramadol daily. The fact that the participants’ distance to their drug use differs may also affect their accounts, as their earlier life experiences are filtered through their current life situation and drug use status (Järvinen, 2000).

All but two of the participants had experiences of interventions from social services and healthcare due to their drug use, such as outpatient care, treatment institutions, foster care, and inpatient psychiatric care. One of them was in opioid substitution treatment. Three had been in compulsory care due to their drug use. Their experiences of treatment ranged from having been continuously enrolled in various forms of interventions since early adolescence, to being in treatment for the first time. Half of the participants mentioned involvement in crime and four were currently on probation.

Only two of the participants lived with their parents. Seven were in residential care, two lived with a partner, and one had supported housing. Three of the participants had come to Sweden as unaccompanied minors. Some of the participants reported a difficult family background, while others emphasised that they have always had a supportive family. Most of them expressed problems with anxiety, and seven had diagnoses such as ADHD and PTSD. A majority of the participants had dropped out of school and none of them were currently enrolled in any studies. Some of them had been working during the period they used drugs, while a few others had started job training after quitting drugs.

## Results

Three central themes emerged through the process of analysis. The first theme—*quitting initiated by parents and professionals*—highlights the role of external pressure and influence to stop using drugs and participate in treatment. The next theme—*being willing, but unable*—illustrates the struggles and difficulty of quitting tramadol highlighted in the young people's accounts. The last theme—*between ambivalence and determination*—concerns the dynamic nature of the participants’ motivation to quit drugs.

### Quitting initiated by parents and professionals

Young people often begin treatment due to pressure and demands from parents and professionals, and commonly lack intrinsic motivation to change their drug use, at least initially (Battjes et al., 2003; Hugo et al., 2021; Wilson & Saggers, 2013). In line with this, many of the participants in the study reported a high degree of external pressure to stop using drugs. Treatment interventions were often initiated immediately after their drug use was first noticed by their parents, staff at school, or other professionals. They describe these interventions as meaningless and a waste of time, but they participated anyway, in an effort to keep responsible adults such as parents and social services calm. At the same time, they usually continued using drugs. Alex offers a typical description:

For me, it was just something I did to make my loved ones a little calmer. Well, I go there once a week then […] She was very good who I talked to there, but it didn’t give me anything. I kept using drugs during the whole thing. And I went there, sure, but it was nothing concrete. We just sat there and talked. And I wasn’t taking it seriously either, I just thought it was bothersome to go there. Then it was more like ‘he smokes weed, he takes some tablets once in a while, it's not that bad yet’. And I also noticed that they thought that way. So, I wasn’t too worried about social services, at least not then.

To agree to treatment could also be a strategy to avoid compulsory care. Linus was told by the social services that “*Either you go there, or it will be compulsory care*”*.* A common strategy was also to hide the extent of drug use, for example by manipulating drug tests. Even residential care did not prevent young people from using drugs, as Max puts it: “*At the first treatment home I lived in, I abused so much tramadol there, so it's surprising that I’m actually alive today*”. Similar to Max, several of the young people describe how parents and professionals tried to make them stop using drugs, but that they were not listening. At the time, they were not concerned about the risks and consequences of their drug use, and their use of tramadol also made them feel careless. Max demonstrates: “*People were having seizures and collapsed and hit their heads. I didn’t give a shit about that, I continued to use anyway*”.

Furthermore, the study's young participants had mainly positive experiences linked to their tramadol use at the beginning and lacked their own incentives for quitting. They emphasised a lack of intrinsic motivation as the main reason for previous treatment failures, sometimes combined with dissatisfaction with the services offered. Johan, who had been enrolled in many different interventions by the social services due to his drug use, explains:

A large part of it depended on me and my attitude too, 100%. I’m not just trying to blame others. There weren’t such nice places [residential care and foster home] I ended up in, but it was because of my own attitude as well. But it could have been such a small thing, that you had ended up in a good foster home, or a place with good staff, who might have been able to change my attitude.

In contrast to this, there was also another type of accounts, where the drug use was unknown to parents and professionals. These young people continued their drug use for a long time, often for several years, without any external pressure to quit. However, sooner or later, their drug use was somehow discovered or they sought help on their own.

While previous interventions were described as meaningless, later on current treatment interventions were highlighted as significant. This was regardless of whether they were initiated by the young people themselves, or externally. Many of them had an ambivalent approach to drugs at the beginning of treatment and claimed that both their motivation and ability to stop using drugs increased during treatment, as was the case for Noel:

I was probably not quite ready, you know, in the mindset when it comes to quitting. When I first came here, I thought ‘I’ll do these three months. Get some rest, gain some weight. And then I’m back on it again.’ But after these relapses, I felt stupid, you know. So now these last few days I’ve felt great, no anxiety … nothing! I feel that ‘Yes, I can live without drugs’. Because it's always been the anxiety that has fucked up my head. That's what made me do drugs, otherwise I shouldn’t have used drugs. The way I feel now, I could feel this way all my life, without using drugs. Because now I feel ready, at last.

The beginning of treatment was often described as ‘messy’, as they continued to use drugs, or had several relapses. They emphasised the importance of treatment staff being patient and not giving up on them, such as Reza comment: “*They really wanted to help me, so they gave me some time, until I got tired myself*”. Even though the treatment itself is not pointed out as crucial for their eventual turning point, it appears to have been supportive and motivating in their process of change. In contrast to earlier interventions, current treatment was described as helpful in many ways, where the relationship with staff being emphasised. Linus says: “*The staff here, they really care about you […] They accept you for how you think and so on, even though it may not be right*”. Some participants pointed out specific treatment staff as particularly significant, for example Najib: “*It is because of him that I am sitting here today […] If there is anything he can do to make me better, he will do it*”. For others, the emphasis is on the relationship to family and friends. Johan explains:

Even though I had been such an asshole and been using drugs, lying, and stealing, they [family and partner] were still by my side. And then I really understood that, well, they truly love me. And then I have to do something now, to show them that I care about them as well. So that was what motivated me the most.

Overall, it appears that the process of quitting drugs was often initially imposed by parents and professionals. Deci and Ryan (2008) claim that external motivation can undermine intrinsic motivation, as it can lead to feeling of being controlled. However, it also appears that trusting relationships with treatment staff or family/friends have had a motivating role for many of the young people. According to Deci and Ryan (2008), feeling connected to other people facilitates the internalisation of values and behaviours that are endorsed by them.

### Being willing, but unable

It appears that the young people in the study largely used tramadol for self-medicating purposes. The relationship between mental health issues and drug use is dynamic, where self-medication can play a role in both inducing and maintaining drug use (Darke, 2012). While mental health issues are widespread among young drug users in general, earlier research shows that it does not appear to be more prevalent among tramadol users specifically (Holmstedt et al., 2020; Olsson et al., 2017). However, opioids in general have a particularly high potential for dependence, with factors such as withdrawal relief and neuroadaptation contributing to a continuous use (Darke, 2012). Quitting opioids can therefore entail extra challenges, in comparison with drugs such as cannabis, as evident in the young people's accounts.

A recurring theme in the young people's accounts was earlier experiences of being unable to stop using tramadol, as they quickly experienced a dependence on the drug. Najib describes: “*You get stuck in it. You have to take it. Otherwise, the body won’t function. In the beginning it was good, but in the end, it was 6–8 tablets every day*.”

Many of the young people emphasised their mental state as the primary reason for their difficulty in quitting the drug, or even made quitting seen as an unrealistic option. Alicia, who used tramadol continuously, describes:

I wish I have had a break, but it didn’t work out when you feel as bad as I did […] If I had one day where I felt like ‘no, I’ll try not to use’. And then the anxiety was getting stronger and stronger and you’re kind of just thinking ‘I have it at home, so why shouldn’t I just take one, so I can feel a little better at least?’ So unfortunately, there were no breaks.

Others, like Damir, had many short breaks in his use of tramadol, but also found reasons to postpone quitting:

I’ve had maybe 30 breaks, but a maximum of 1 week. Once maybe a month, a maximum of 1 month. There have been short breaks where I’ve tried, and then I’ve thought that ‘I just take so little, I can stop later, can stop later, can stop later’.

Many of the participants highlighted mental health issues as their main problem but claimed that they have not received any help with this and were not taken seriously by psychiatry. Noel says: “*They see me as an addict, then you don’t get any help*”; and explains that: “*You have to be clean for up to 3 months before you get any help. Then I have felt like, I can’t do it*”. Furthermore, some of the young people report needing the drug in order to manage social interaction and experienced difficulties in social situations when trying to quit tramadol. Damir says: “*It feels almost like you have social phobia when you stop taking it*”.

Another barrier for quitting tramadol that is highlighted is the withdrawal symptoms. This is described as a terrifying experience that was so hard to bear that they often ended up taking the drug again. This is highlighted as an extra challenge in quitting tramadol, compared to cannabis or other drugs that do not cause withdrawal symptoms in the same way. As Alex explains:

When I went off cannabis, I was sweating during sleep and had a lot of nightmares and so on. But when I went off tramadol … every time I tried to get off it, it was like this; I was vomiting, I was like ‘dope sick’. I got like the flu, but 1000 times worse and I felt like a disaster. And at those times I didn’t have enough will to quit, so I just took it again to be able to survive, it felt like. I think that physically, it's probably the worst thing I’ve experienced.

As Alex described, it appears that the combination of the withdrawal symptoms and ambivalence towards the drug use can create a vicious circle. It is very difficult to manage something so tough if you are not determined enough to quit. Some of the participants also had relapses after periods of abstinence, where poor mental health in combination with the availability of drugs among their circle of friends being identified as the primary risk factors. Saga relates her recent relapse on tramadol:

I’ve had it around me. So that's what caused it, that it was available. And I didn’t feel well, and I know that it helps. So, it just happened. But then I continued a few times, because there is such a big difference in my mood. I took tramadol and I felt great, could be out among people and all problems disappeared, all my anxiety and such. And then, when I was no longer affected, I realised that ‘yes, I feel quite shitty’. And I know, if I take another one, it's gone. So, it happened a few times.

Another common context for relapse was to return to their hometown after treatment. Doubts about their ability to abstain from drugs when returning was particularly clear among the young people who had come to Sweden as unaccompanied minors. They described how they were forced to leave treatment before they felt ready, due to decisions made by social services, like Reza:

This is my second time in treatment. Last time I was there for a year and during that year I stayed away from drugs and alcohol for 8 months. Then they moved me back to [hometown]. And I told them, ‘I’m not ready’ and so on. ‘If I move to [hometown], I will meet those people. I’m not sure that I can say no to drugs’. They said, ‘We think you can say no’. Then they moved me back and I ended up on drugs again.

Overall, the young people expressed earlier experiences of inability to quit tramadol and abstain from drugs, due to dependence, mental health issues, and withdrawal symptoms, in combination with the availability of drugs. This is what Ryan and Deci (2017) refer to as lack of *competence*, which can be an obstacle in making important life changes. However, it appears that over time, many of them have improved their ability to resist cravings and manage emotional distress without using drugs.

### Between ambivalence and determination

Ambivalence can be described as an internal conflict between two courses of action, where both options have advantages and disadvantages (Rodriguez & Smith, 2014). Previous studies have shown that “drug life” is often characterised by feelings of ambivalence towards drug use, which can make it difficult to successfully quit and maintain that change (Blomqvist, 2002; Kristiansen, 1999; Skårner, 2001). Ambivalence is constantly present in the accounts of the young people, who are torn between their love for the drug and the negative consequences it entails. Anton explains: “*It's like there's a devil and an angel on your shoulders. One says, ‘take it’, the other says ‘do not take it’. But you always listen to the evil one*”. In the beginning, their tramadol use was mainly associated with positive, euphoric experiences. Several of the young people describe that they used tramadol to self-medicate, as the drug made anxiety disappear and social situations easier to handle. Furthermore, they had positive experiences linked to its activating effects, such as being able to work hard or keep up concentration at school. The drug use was also associated with fun memories with friends, as Johan puts it:

I wanted to quit. Because, well, it did not bring much good stuff with it. There were quarrels in the family and they were disappointed and sad and worried and everything. But then it was also the thing that … it was what we were doing, me and my friends. We were hanging out, taking tramadol, smoking weed and playing football. We actually had a lot of fun together. We did a lot of fun stuff. But it was just that … in order to do these fun things, we took tramadol and smoked. So, at the same time … in one way I wanted to quit, but at the same time I missed hanging out with my friends and having as much fun as we used to.

Eventually, however, their use of tramadol and other drugs began to have a negative impact on various areas of life, such as health, school, and relationships. This was often associated with the usage of higher and higher doses of tramadol due to increased tolerance. Some of them reported daily doses in the range of 1000–3000 mg, significantly higher than the recommended maximum dose of 400 mg when the drug is prescribed (Boostani & Derekhsan, 2012). Most of the participants mentioned physical side effects such as stomach problems, loss of appetite, vomiting, and severe weight loss. Noel says: “*It feels like the body is kind of rotting*”. In contrast, others indicated that they only felt bad when they did not take the drug.

Another reported side effect from tramadol was seizures. Some of the young people have had seizures themselves, while others had witnessed friends having seizures. There were also experiences of negative emotional impact from tramadol. Emil states: “*My mother had not heard me laugh in 3 years. Not a laugh. It completely shuts off your whole personality*”. Despite negative consequences, there were also factors that pulled them back to drugs, as Reza explains:

You wake up one day and have no money. And you sit there thinking ‘Fuck, I have to quit with this shit’. Then you stay away from drugs for maybe a few days … then it starts to feel stressful … and you have found money … and when you walk on the street, everything is there … drugs today, it is everywhere. So, it's the person himself who has to decide. And it takes a little time, it's not happening just like that.

The young people also expressed conflicting feelings when it comes to seeking help for their drug problem. Some had postponed or avoided seeking help due to a lack of trust in social services. This was the case even if they already had contact with social services for other reasons. Damir, for example, had not told social services about his tramadol use and says: “*No, I don’t dare, because maybe they say something like ‘You have to leave negative drug tests to get money’ or something*”. The hesitation to seek help is also explained by the perception of being self-sufficient, as Hossein declares:

When you accept

Okay, I’m a junkie”, then you will start asking for help. But I couldn’t accept this thing that I’m a junkie, you know. I always thought: “I have always managed alone in all situations […] I will not take help from anyone. I can take care of myself, I can stop. I can do drugs whenever I want, but I can stop whenever I want.

However, the attitude of “managing oneself” could also be an expression of a lack of trust. For example, Alicia explains that her self-reliant attitude and skepticism toward psychiatry and social services stem from an experience of not being cared for. She says: “*Nobody cares to help me anyway. Everyone does it for their own benefit in the end*”.

It appears that most of the young people, sooner or later, reached a point where they made a clear decision to quit drugs. Some spoke of a turning point that was related to a specific event or episode where their drug use escalated, such as Alex, who had several tramadol-induced seizures in a short period of time:

My parents came and picked me up, and we went to the hospital. Then, my parents said, ‘We can’t stand this anymore, this is the last time we drive you here’. And the doctor looked at me and then looked in his medical record and said, ‘Now you have been here quite a few times in a short time’. And that's not so good [laughs], those seizures are not that healthy. Then he said that ‘You will die if you continue now’. So, I got a kind of a wake-up call there, it became quite obvious that the body couldn’t manage this anymore.

In contrast, others had a more general experience that the drug use had gotten out of control, like Alicia: “*December 2019. Then I was taking up to 4–5 [tramadol] a day, just to stay up. And that was when I felt like, this is chaos, this isn’t working*”. There were also those who describe more of an inner realisation that change was necessary and compared themselves to peers who have progressed further in life, as Max put it: “*I’ve lost all these years, you know. Everyone is out making money and I’m sitting here doing drugs. I can no longer stand it*”. Some also spoke of positive events that contributed to a turning point, such as a relationship with a partner, or an experience of no longer needing drugs.

In most of the young people's accounts, there was a clear distinction between their experiences “before” and “after” they decided to quit drugs. The past was associated with ambivalence, impulsivity, and carelessness, while the present was associated with increased awareness and a personal choice to abstain from drugs. In other words, they expressed increased autonomous motivation, even though ambivalent feelings still remained. Deci and Ryan (2008) suggest that motivation is largely related to sociocultural conditions, where autonomy support from significant others is of great importance. In drug treatment, autonomy-supportive techniques include an empathetic and non-judgemental approach that allows the individual to safely explore their ambivalence and resistance to change (Ryan & Deci, 2017).

## Discussion

The present study sought to explore young people's experiences of trying to quit drugs, focusing on the non-medical use of tramadol. The findings of the study demonstrate conflicting feelings about drug use, external pressure, and influence, as well as earlier experiences of inability to quit. However, many of the participants reported an increased willingness and ability to abstain from drugs over time. This can be linked to a psychosocial context which has promoted their needs of *autonomy*, *competence*, and *relatedness* (Deci & Ryan, 2008). Table 1 illustrates this. It should be noted that it is not a matter of going from one position to another, but rather taking steps in that direction. In addition, the young people are still in the midst of an ongoing process, whose continued direction remains uncertain.

**Table 1.** Study results, according to self-determination theory.

Social context	Autonomy	Competence	Relatedness
Hindering	Insufficient inner will to change drug use, along with significant external pressure to quit. High degree of ambivalence.	Feelings of being unable of quitting drugs. Experiences of needing drugs to feel good or function normally.	Lack of trusting relationships. Sense of relatedness primarily tied to drug-using friends.
Facilitating	Increased intrinsic motivation to quit drugs. Abstaining from drugs as their own genuine choice.	Do no longer need drugs to feel good. Have other strategies to deal with emotional distress.	Trusting relationships with treatment staff and/or family/friends. To be cared of and be significant to others.

The findings show that many of the young people in the study have experienced significant external pressure to stop using drugs and undergo treatment, consistent with previous research (Battjes et al., 2003; Hugo et al., 2021; Wilson & Saggers, 2013). However, these interventions did not give the desired results due to a lack of autonomous motivation and a social environment that did not support this. On the other hand, for some, drug use was hidden from parents and professionals for years and there was neither external nor internal motivation to quit.

The young people's accounts reflect their ambivalent feelings about drug use, but also a gradual increase in their intrinsic motivation to quit. This is partly linked to the growing negative consequences of drug use, as previous studies have shown (Battjes et al., 2003; Goodman et al., 2015). Another motivating factor, also noted in other studies, is a sense of falling behind in life, regarding aspects like employment and economic status (Nehlin et al., 2020; Søgaard et al., 2016). Treatment interventions also appear to have played a significant motivational role for many of the young people. In line with previous studies, the initial part of treatment was the most critical phase, marked by ambivalence and relapses (Nash et al., 2015; Rodriguez & Smith, 2014;). However, eventually they reached a turning point, where supportive treatment staff who did not give up hope were crucial. This can be seen as an internalisation process, where the young people, with support from a facilitating social context, changed their approach to drug use and developed autonomous motivation to change (Deci & Ryan, 2017).

The Nordic countries, with their “generous welfare state” provide greater opportunities to get help from society to leave criminality and drugs, compared to many other countries (Søgaard et al., 2016). Nevertheless, the young people emphasised their own willpower and achievement, rather than contextual factors. This is consistent with previous Nordic studies that have revealed an “individualized responsibilization discourse” in residential care for young people (Gradin Franzén, 2015; Søgaard et al., 2016).

The findings also demonstrate the challenges the young people face in quitting tramadol, which Ryan and Deci (2017) refer to as a lack of *competence.* The participants reported that emotional distress and withdrawal symptoms contributed to their continued tramadol use and some of them argued that they had experienced a stronger dependence on tramadol compared to other types of drugs. Opioid use is associated with higher dependence liability, substance use severity, and poorer long-term prognosis compared to other types of drugs (Darke, 2012; Subramaniam & Stitzer, 2009). Darke (2012) describes how self-medication interacts with factors such as withdrawal relief and neuroadaptation and contributes to the maintenance of the dependence. Similar to other studies, the young people reported multiple attempts to quit that failed, or where they ended up leaving one drug for another (Nehlin et al., 2020). Mental health issues combined with high access to drugs in their social environment were identified as the main risk factor for relapse. Previous studies have shown that relapse among young people often occurs in a social context among drug-using friends (Nash et al., 2015; Rodriguez & Smith, 2014). However, it also appears that most participants have improved their ability to resist drug cravings and manage without drugs over time.

The importance of relatedness is also clear in the young people's accounts. It appears that their sense of relatedness was previously primarily tied to drug-using friends. Earlier treatment interventions were often associated with a lack of trusting relationships and avoidance of treatment efforts was often linked to lack of trust in social services. In contrast, current treatment interventions were associated with trusting relationships with people who genuinely cared about them and whom they really listen to. These different experiences can be partly explained by the fact that they have been at different stages of their change process. However, trusting relationships per se can also promote an increased motivation for change (Hugo et al., 2021). Ryan and Deci (2017) claim that people are more likely to adopt values and goals from people whom they feel connected to or can identify themselves with. In addition to professionals, supportive partners, friends, and family also played an important role for several of the young people.

This article highlights the importance of trusting relationships with people who do not give up hope, both inside and outside treatment. Young people are rarely fully motivated to stop using drugs initially when entering treatment and cannot be expected to be either (Nash et al., 2015). It is time to abandon the notion that people must be “done doing drugs” before they are ready to start treatment (Johnson et al., 2017). Instead, social services and healthcare providers need to pay attention to how best to meet the often ambivalent attitudes of young people towards drug use and treatment. There is a need to understand both their positive and negative experiences associated with drug use and help them reflect on this without inducing guilt or condemnation. Furthermore, it is important to provide an environment that supports their basic needs for autonomy, competence, and relatedness (Ryan & Deci, 2017). Social services and healthcare providers play a crucial role, not only in offering treatment interventions that match the individual's needs, but also in creating trust.

Furthermore, there is a need for awareness of the difficulties associated with quitting tramadol use. The study highlights the challenges faced by young people in attempting to stop using the drug, including experiences of dependence, withdrawal symptoms, and mental health issues. The study participants' drug-related problems also appear to be more severe than what is commonly associated with young people. The probems resembled more those faced by people longer ahead in their drug careers. This may be due to the fact that these young people initiated regular opioid use at a relatively young age through their use of tramadol. Opioid dependence poses a risk of harm to the individual that by far exceeds all other types of drugs (Darke, 2012). Furthermore, it appears that several of the young people eventually transitioned from tramadol to stronger opioids, such as heroin. Non-medical use of prescription opioids is considered a strong risk factor for initiating heroin use, which carries additional severity and risks (Compton et al., 2016; Guarino et al., 2018; Martins et al., 2017). Difficulties reaching young tramadol users with effective treatment interventions might therefore also entail the risk of an increasing number of young people using heroin.
